# Predictive factors of pathologic complete response in HER2-positive and axillary lymph node positive breast cancer after neoadjuvant paclitaxel, carboplatin plus with trastuzumab

**DOI:** 10.18632/oncotarget.17993

**Published:** 2017-05-19

**Authors:** Jinhua Ding, Yinlong Yang, Li Jiang, Weizhu Wu, Zhiming Shao

**Affiliations:** ^1^ Department of Breast and Thyroid Surgery, Ningbo Medical Center Lihuili Eastern Hospital, Ningbo, China; ^2^ Department of Breast Surgery, Cancer Center and Cancer Institute, Shanghai Medical College, Fudan University, Shanghai, China; ^3^ Department of Emergency, Ningbo Medical Center Lihuili Eastern Hospital, Ningbo, China

**Keywords:** breast cancer, neoadjuvant chemotherapy, human epidermal growth factor receptor 2 (HER2), Ki67, pathological complete response (pCR)

## Abstract

**Objective:**

This study was performed to investigate the proportion as well as the predictive factors of pathologic complete response in HER2-positive and axillary lymph node positive breast cancer after neoadjuvant paclitaxel, carboplatin plus with trastuzumab (PCH).

**Results:**

The pCR rate in the breast, axilla and both was 44.3% (39/88), 47.7% (42/88) and 34.1% (30/88), respectively. Patients with and without pCR were similar in term of age, BMI, menstrual status, family history, treatment cycles and tumor characteristics (laterality and size of tumor). Multivariate logistic regression demonstrated that pCR was significantly associated with HR negativity (HR = 5.587, 95% CI 2.25−3.889, *p* < 0.001), high Ki67 index (HR = 4.130, 95% CI 1.607−10.610, *p* = 0.003). Further investigation found that patients with HR-negative/high Ki67 index had higher pCR rate, compared to other patients (HR = 7.583, 95% CI 2.503−22.974, *p* < 0.001).

**Materials and Methods:**

88 consecutive Chinese HER2-positive/axillary lymph node-positive breast cancer patients with neodjuvant therapy regimen containing paclitaxel, carboplatin and trastuzumab were divided into two groups: pathological complete response (pCR) or non-pCR group. Clinico-pathological characteristics were compared and analyzed, and univariate and multivariate analyses were performed to detect the predictive factors of pCR.

**Conclusions:**

Preoperative PCH regimen was an effective neoadjuvant therapy in HER2 positive and axillary lymph node positive patients, and patients coexisting with HR-negative and high Ki67 index may benefit more from this regimen.

## INTRODUCTION

Amplification of HER2 is identified in approximately 20–25% of human breast cancers [1, 2]. The combination of a chemotherapeutic regimen and a recombinant monoclonal antibody against HER2, trastuzumab, has formed the cornerstone of therapy for HER2-positive breast cancer. The superiority of the addition of trastuzumab to a taxane-based chemotherapy regimen is supported by data from metastatic, neoadjuvant and adjuvant studies [3–5]. However, the pCR rate of neoadjuvant chemotherapy (NAC) using taxanes and trastuzumab was not high, ranging from 20 to 40% in early studies [6, 7]. Carboplatin, instead of anthracyclines, has also been implemented to NAC regimens in an attempt to obtain a higher pCR rate, and indeed, this regimen containing taxane, carboplatin and trastuzumab has been confirmed to be effective in HER2 positive disease [8–10].

However, compared to lymph node negative breast cancer, tumor with HER2 positive and lymph node-positive was a subtype of biological and anatomic more aggressive disease. The effectiveness of this regimen used for this type of disease was not established, and the predictive factors of pCR have not been clarified yet. Thus, in our study, data of patients with HER2-positive and lymph node positive disease, who were treated with carboplatin, paclitaxel concurrently with trastuzumab was retrospectively analyzed, in order to identify effective predictive markers for pCR that could be used in the next future for treatment decision making.

## RESULTS

### Patients’ characteristics

Between July 2010 and December 2014, a total of 88 HER2-positive patients with breast cancer were enrolled in the present cohort. Median age was 52 years (ranging from 21 to 71). The proportion of patients aged than 35 years and the opposite was 85.2% and 14.8%, and that with the status of premenopausal and postmenopausal were 48.9% and 51.1%.

### Neoadjuvant cycles and surgery

After two cycles of neoadjuvant chemotherapy, 6 patients were transferred to surgery because of SD, and another 6 patients were also transferred to surgery owing to their own willing, despite of CR or PR. After four cycles, another 30 patients were transferred to surgery on account of SD. Finally, 46 patients finished six planned cycles of neoadjuvant chemotherapy.

In spite of high clinical response, the majority of patients still had an obstinate conception that modified radical mastectomy was much safer than breast conserving surgery, so finally only 8 patients received breast conserving surgery. Axillary lymph node clearance were performed in all of 88 patients. Table 1 presents the patients’ demographics and clinicopathologic characteristics.

**Table 1:** Demographics and clinicopathologic characteristics of 88 patients

	*n*	%
Case number	88	
Mean age (years)	52	
Menstrual status		
Premenopause	43	48.9
Postmenopause	45	51.1
Age		
≤ 35 years	13	14.8
> 35years	75	85.2
BMI		
≤ 25	54	61.4
> 25	34	38.6
Laterality		
Left	50	56.8
Right	38	43.2
Family history		
Yes	6	6.8
No	82	93.2
Number of cycles		
< 4	12	13.6
≥ 4	76	86.4
ER status		
Negative	46	52.3
Positive	42	47.7
PR status		
Negative	54	61.4
Positive	34	38.6
HR status		
Negative	44	50.0
Positive	44	50.0
Ki-67		
High (> 30%)	39	44.3
Low (≤ 30%)	49	55.7
Primary tumor stage		
T1	14	15.9
T2	59	67.0
T3	13	14.8
T4	2	2.3
Clinical Response		
CR	20	22.7
PR	62	70.5
SD	6	6.8
PD	0	0
Surgery type		
Breast conserving surgery	8	9.1
Mastectomy	80	90.9

BMI: body mass index; ER: estrogen receptor; PR: progesterone receptor; HR: hormone receptor; T: tumor;

CR: complete response; PR: partial response; SD: stable disease; PD: progression of disease.

### Efficacy evaluation

Results of clinical response evaluation are also demonstrated (Table 1). There was no PD in 88 eligible patients, and CR, PR, SD were achieved in 20, 62 and 6 patients, respectively. The overall clinical response rate (clinical CR and PR) was 93.2% (82/88).

As for pathological response, pCR in the breast was achieved in 39 patients (44.3%), in the axilla in 42 patients (47.7%), and in both position in 30 patients (34.1%). The pathological response evaluation after neoadjuvant treatment is described in Table 2.

**Table 2:** Pathologic tumor characteristic after neoadjuvant chemo-trastuzumab therapy

Category	*N*	%
Pathological tumor category		
ypT0	37	42.0
ypTis	2	2.3
ypT1	22	25.0
ypT2	24	27.3
ypT3	3	3.4
Pathological nodal category		
ypN0	42	47.7
ypN1	23	26.1
ypN2	14	15.9
ypN3	9	10.2
Combination tumor and nodal category		
axillary pCR+ / tumor pCR+	30	34.1
axillary pCR+ / tumor pCR−	12	13.6
axillary pCR− / tumor pCR+	9	10.2
axillary pCR− / tumor pCR−	37	42.0

pCR: pathologic complete response; T: tumor; N: node.

### Clinicopathological factors of pCR rate

Significant clinicopathological factors that affect pCR were identified and compared between the two groups in Table 3. The pCR was more commonly detected with HR-negativity (50.0% vs 18.2%, *p* = 0.002). The pCR group showed more cases with high Ki67 index (48.7% vs 22.4%, *p* = 0.013).

**Table 3:** Relationship of clinicopathologic factors for pCR

Variabe	pCR (*n*)	Non-pCR (*n*)	*P* value	Hazard ratio	95% CI
Menstrual status					
Premenopause	13	30	0.455	0.714	0.294–1.733
Postmenopause	17	28			
Age					
≤ 35 years	4	9	0.784	0.838	0.235–2.983
> 35years	26	49			
BMI					
≤ 25	20	34	0.462	1.412	0.562–3.548
> 25	10	24			
Laterality					
Left	14	36	0.167	0.535	0.219–1.305
Right	16	22			
Family history					
Yes	3	3	0.406	2.037	0.385–10.770
No	27	55			
Cycles					
< 4	4	8	0.952	0.962	0.265–3.494
≥ 4	26	50			
HR status					
Negative	22	22	0.002	4.500	1.710–11.841
Positive	8	36			
Ki-67					
High (> 30%)	19	20	0.013	3.282	1.309–80227
Low (≤ 30%)	11	38			
T stage					
T1–2	24	49	0.596	0.735	0.234–2.303
T3–4	6	9			

pCR: pathologic complete response; BMI: body mass index; HR: hormone receptor; T: tumor; CI: confidence interval.

### Predictive factors of pCR rate

Multivariate logistic regression analysis including all parameters which were analyzed in univariate analysis showed that HR negativity and high Ki67 index were two independent pCR predictors (Table 4). The hazard ratios for HR negativity and high Ki67 index was 5.587 (95% CI, 2.257–13.889) and 4.130 (95% CI, 1.607–10.610), respectively.

**Table 4:** Multivariate analysis for pCR predictive parameters

Variable	pCR (*n*)	Non-pCR (*n*)	*P* value	Hazard ratio	95% CI
Menstrual status					
Premenopause	13	30	0.191	2.391	0.647–8.831
Postmenopause	17	28			
Age					
≤ 35 years	4	9	0.131	4.168	0.655–26.532
> 35years	26	49			
BMI					
≤ 25	20	34	0.432	1.558	0.516–4.708
> 25	10	24			
Laterality					
Left	14	36	0.121	2.035	0.830–4.993
Right	16	22			
Family history					
Yes	3	3	0.086	0.441	0.173–1.123
No	27	55			
Cycles					
< 4	4	8	0.897	1.102	0.253–4.806
≥ 4	26	50			
HR status					
Negative	22	22	< 0.001	5.587	2.257–13.889
Positive	8	36			
Ki-67					
High (> 30%)	19	20	0.003	4.130	1.607–10.610
Low (≤ 30%)	11	38			
T stage					
T1–2	24	49	0.780	0.850	0.272–2.659
T3–4	6	9			

pCR: pathologic complete response; BMI: body mass index; HR: hormone receptor; T: tumor; CI: confidence interval.

### pCR rate on subgroups defined by predictive factors

Correlation analysis shows that Ki67 index did not correlate to HR status (coefficient was 0.025, *p* = 0.820). Thus, based on the status of HR and Ki67 index, 88 patients were classified into four groups: HR-negative/low-Ki67-index group (*n* = 24), HR-negative/high-Ki67-index group (*n* = 20), HR-positive/low-Ki67-index group (*n* = 25), HR-positive/high-Ki67-index group (*n* = 19).

The pCR rate between subgroups stratified according to HR status and Ki67 index is demonstrated in Table 5. The pCR rate in HR-negative/high-Ki67-index group (70.0%, 14/20) was higher than in the other groups(23.5%, 16/68), the hazard ratio was 7.583 (95%CI, 2.503–22.974, *p* < 0.001).

**Table 5:** Comparison of pCR rate between groups defined by hormonal receptor and Ki67

Group	Definition	Case number	pCR (*n*)	Non-pCR (*n*)	pCR (%)
1	HR negative and low Ki67 index	24	8	16	33.3
2*	HR negative and high Ki67 index	20	14	6	70.0
3	HR positive and low Ki67 index	25	3	22	12.0
4	HR positive and high Ki67 index	19	5	14	26.3

*Group 2 was compared with the combination of Group 1 + Group 3 + Group 4.

*p* < 0.001, HR = 7.583, 95% CI, 2.503–22.974.

## DISCUSSION

To the best of our knowledge, this is the first and sole study to identify the predictive factors for pCR on HER2-positive/axillary lymph node positive breast cancer patients receiving neoadjuvant regimen containing paclitaxenl and carboplatin concurrently with trastuzumab. In the present study, we clarified that the NAC regimen with paclitaxenl-carboplatin-trastuzumab was useful for obtaining a pCR rate of 34.1%. The pCR rate in our study was much lower than in previous reports, in which the similar regimen was adopted and achieved the pCR varying from 44–76% [8–10]. Significant difference between our study and others must be stated that the cases enrolled in our study were axillary lymph node positive, while the cases in others comprised of both lymph node positive and negative disease. In addition, the majority of tumors in our study were T2 or even higher stage. Both factors may contribute to relative low pCR rate in our study.

The predictive value of HR status for pCR after trastuzumab-based neoadjuvant was controversial, though positive results were found in the majority of the studies [11–16]. While, HR status was not demonstrated a predictive factor for pCR in other studies [17, 18]. In above studies, carboplatin was not contained in neoadjuvant regimens. Our study confirmed that HR status was an independent predictive factor for pCR after neoadjuvant regimen containing paclitaxel, carboplatin and trastuzumab, which has been repetitively reported recently [19–21]. However, the neoadjuvant regimen was different in these studies, including DC-H (docetaxel, carboplatin, trastuzumab) or D-H [19], DC-HP (docetaxel, carboplatin, trastuzumab, pertuzumab) [20], PC-H (paclitaxel, carboplatin, trastuzumab [21].

The second independent predictive factor detected in our study was Ki67 index. The Ki67 cut-off point in different studies varied between 5% and 34% [22–24], and the ideal cut-off point has not come to a consensus yet. The predictive value was validated in the majority of previous studies [19, 25–29]. However, another retrospective study in which the same neoadjuvant regimen was administrated as that in our study, showed that high Ki67 index was not associated with higher pCR [21]. The controversial result between this study and ours may result from the different baseline characteristics of enrolled patient population, because all patients in our study were axillary lymph node positive, while the patients in the other study comprised of both node positive (59.4%) and node negative (40.6%) patients.

In our study, further correlation analysis showed that Ki67 did not correlate to HR status, which enabled us to stratify patients into four subgroups based on HR status and Ki67 index: HR-negative/low-Ki67-index, HR-negative/high-Ki67-index, HR-positive/low-Ki67-index, HR-positive/high-Ki67-index group. The pCR rate in HR-negative/high-Ki67-index group was significantly higher than the other subgroups (shown in Table 5), suggesting that combination of HR status and Ki67 index can effectively discriminate patients with differential opportunity of achieving pCR.

The highlight of our study should be recognized. Firstly, patients enrolled in our study were HER2-positive invasive breast cancer with lymph node involvement, which was a subtype of biological and anatomic aggressive disease. In addition, the positivity of axillary lymph node was ascertained by fine-needle aspiration before neoadjuvant therapy, which enabled us to evaluate the pCR in the axillary lymph node after neoadjuvant therapy. Secondly, the neoadjuvant regimen in our study was non-anthracyclines, which can further decrease heart risks. Because chemotherapy drugs, especially anthracyclines can lead to irreversible heart damage, and anti-HER2 drug, trastuzumab may decrease cardiac function in some patients. Thirdly, the neoadjuvant regimen adopted in our study was PCH (paclitaxel, carboplatin and trastuzumab) , which was confirmed to be effective on prior studies, and equally effective to EC-PH (epirubicin, cyclophosphamide, followed by paclitaxel and trastuzumab) [8–10]; while in the other study, the majority of patients (83.3%) were administered with DH or PH (docetaxel or paclitaxel with trastuzumab) regimen, which was applicable for low recurrence risk patients, especially for node negative patients.

Of course, the limitations of the present study also should be acknowledged. First, this was a retrospective, single-institution study with small sample size, which may decrease the reliability of the present study. Second, some parameters, for example, tumor-infiltrating lymphocytes and tumor histological grade before neoadjuvant therapy were not provided, which were proven to be predictive factors in other report [21], and could not be further verified in our study. Third, because of the short term follow-up time, survival data are unavailable in the study. Therefore, it is unclear whether the pCR improvement would transfer to a survival benefit.

## MATERIALS AND METHODS

### Patient selection

Totally 88 HER2-positive breast cancer patients treated with taxane, platinum, trastuzumab preoperatively at Fudan University Shanghai Cancer Center were retrospectively analyzed. Other inclusion criteria included the following: (1) All patients were histologically proven to be invasive ductal carcinoma, (2) At least one axillary lymph node in each patient was proven positive by fine needle aspiration, (3) Status of ER, PR, HER2 and expression of Ki67 were essentially determined before neoadjuvant therapy. Patients either with inflammation breast cancer or with any evidence of distant metastasis were excluded from the study. All patients signed an informed consent before neoadjuvant treatment started.

### Evaluation of ER, PR, HER2 and Ki67

According to the revised version of immunohistochemical testing guideline in 2010, ER and PR assay should be considered positive if immunostaining was seen in more than 1% tumor nuclei [30]. The criteria of HER2 positivity was defined as either IHC3+ or gene amplification by fluorescence *in situ* hybridization (FISH) [31]. In the present study, 30%, the median Ki67 index was used to categorize patients into low Ki67 index group and high Ki67 index group. If a patient had a Ki67 index less or equal to 30%, the patient was considered to have a low Ki67 index, while a patient with a Ki67 index greater to 30% was considered to have a high Ki67 index.

### Treatment details

The treatment regimen including paclitaxel, carboplatin and trastuzumab (abbreviated as PCH) were administrated to 83 patients. The remaining 5 patients received the equivalent of PCH regimen, with nab-paclitaxel instead of paclitaxel in 2 patients and cisplatinum instead of carboplatin in 3 patients, in whom the count of blood platelet was less than 80 billions per litre, and carboplatin using may further decrease the count of the platelet. Treatment cycles were decided by physicians according to the outcome of clinical response evaluation at every two cycles. Six cycles were planned for patients in whom tumor response evaluation was clinical effective, including complete response and partial response. While, the evaluation of stable disease or progression of disease always led to the termination of neoadjuvant therapy and surgery could be performed afterwards.

Breast surgery including extended lumpectomy and axillary lymph node clearance were performed at 2–4 weeks after the last dose. As it should be, trastuzumab with duration of one year in total was recommended for all patients. In addition, adjuvant radiotherapy and endocrine therapy were administered in appropriate patients according to current available clinical practice guideline.

### Data collection

The following data was collected, including: patient's demographics (age, menstrual status, BMI, family history defined as there is one or more breast cancer sufferers in a patient's first or second relatives), pre-treatment tumor characteristics (tumor size, tumor laterality and status of ER, PR, HER2, Ki67 expression), treatment details (drug, dose, cycles, side effect), clinical and pathological response evaluation after neoadjuvant therapy.

Clinical response in the breast and axilla were evaluated using breast ultrasound. Clinical response was categorized as the following: complete response (CR), partial response (PR), stable disease (SD), and progression of disease (PD) [6]. pCR in the breast was defined as disappearance of residual invasive disease (residual ductal carcinoma *in situ* allowed) in the breast by pathologic examination, and pCR in the axilla was defined as the absence of positive lymph node by hematoxylin and eosin staining.

### Statistical analysis

Univariate analysis with chi-squared test or Fisher's exact test was performed to detect predictors for pCR. Multivariate analysis was performed to test factors’ independence by logistic regression analysis. Statistical significance was defined as *p* < 0.05, and hazard ratio (HR) and 95% confidence intervals (CI) were also calculated. All statistical tests were two-sided, and all analyses were performed using SPSS v.19.0 software (SPSS, Chicago, IL, http://www.spss.com).

## CONCLUSIONS

In summary, our study shows that HR negativity and high Ki67 index are two independent factors for predicting higher pCR in HER2-positive/axillary lymph node positive breast cancer. Especially in patients with HR-negative/high-Ki67-index, the pCR rate even reached 70.0%, suggesting that such patient population may achieve more benefit from neoadjuvant therapy containing paclitaxel, carboplatin and trastuzumab.

## Abbreviations

HER2: human epidermal growth factor receptor 2
pCR: pathologic complete response
NAC: neoadjuvant chemotherapy
BMI: body mass index
ER: estrogen receptor
PR: progesterone receptor
HR: hormone receptor
CR: complete response
PR: partial response
SD: stable disease
PD: progression of disease
HR: hazard ratio
CI: confidence intervals
